# The Effect of Formulated Dentin Remineralizing Gel Containing Hydroxyapatite, Fluoride, and Bioactive Glass on Dentin Microhardness: An *In Vitro* Study

**DOI:** 10.1155/2024/4788668

**Published:** 2024-08-10

**Authors:** Mohadese Asadi, Sara Majidinia, Hossein Bagheri, Melika Hoseinzadeh

**Affiliations:** ^1^Dental Materials Research Center, Mashhad University of Medical Sciences, Mashhad, Iran; ^2^Dental Research Center, Mashhad Dental School, Mashhad University of Medical Sciences, Mashhad, Iran

## Abstract

**Objectives:**

This study aimed to develop a gel with dentin-remineralizing properties, integrating nano-hydroxyapatite (nHA), sodium fluoride (NaF), and bioactive glass (BG).

**Materials and Methods:**

The enamel layer of 40 bovine incisors was removed. The samples were allocated into four groups of 10 each, based on varying concentrations of nHA, BG, and NaF in the gel compositions (wt%): (1) 2.5%–7.5%−0.05%, (2) 5%−5%−0.05%, (3) 7.5%–2.5%−0.05%, and (4) a control group with a base gel lacking remineralizing agents. After 8 hr of demineralization, the dentin surface microhardness was measured at depths of 30, 60, and 140 *µ*m. After a 20-day pH cycling, the percentage of surface microhardness recovery (SMHR%) was measured and compared among the groups using the ANOVA and Tukey HSD post hoc tests (*α* = 0.05). Scanning electron microscopy analysis evaluated each specimen's superficial morphology.

**Results:**

At all depths, the SMHR% of the Group 2 and Group 3 was significantly higher than the control group (*p*  < 0.05). The SMHR% Group 1 (67.39% ± 29.34%) was significantly higher than the control group (−21.24% ± 51.72%) only at the depth of 30 *μ*m (*p* = 0.047). Group 3 had higher SMHR% than Group 2 at all depths; however, the difference was not statistically significant. Moreover, the SMHR% of Group 3 was significantly higher than that of Group 1 at depths of 30 *μ*m (187. 94% ± 68.95% vs. 67.39% ± 29.34%; *p* = 0.005) and 60 *μ*m (179.55% ± 75.96% vs. 64.34% ± 41.96%; *p* = 0.043). Surface deposition and tubule occlusion were observed in the Groups 2 and 3 samples, which was more prominent in the latter.

**Conclusions:**

Combining 7.5% nHA, 2.5% BG, and 0.05% NaF could potentially remineralize primary carious lesions.

## 1. Introduction

Untreated dental caries is the most common chronic disease globally [1]. Cariogenic bacteria generate organic acids, like lactic acid, which dissolve and reduce the mineral content of hydroxyapatite (HA) crystals [2]. In minimally invasive dentistry, one of the critical advancements has been the focus on preserving demineralized dentin [3]. Dentin remineralization includes the creation of a new mineralized collagen matrix and the development of hydroxyapatite crystals, which is more complex than the remineralization of enamel [4]. However, most studies have focused on enamel remineralization [5].

Fluoride therapy, widely used for hard tissue remineralization and preventing enamel wear as well as the formation and progression of white spot lesions (WSLs), is effective, but its impact generally reaches only the first 10–30 *µ*m of the lesion [6, 7, 8, 9, 10, 11]. This limitation has led to the development of new strategies for remineralizing deeper lesions, including the use of casein phosphopeptide-amorphous calcium phosphate (CPP-ACP), HA, and bioactive materials such as bioactive glass (BG) [12, 13].

BG is known for its role in dentin remineralization, primarily by blocking dentin tubules by depositing calcium phosphate on the surface [3]. With its high calcium content and rapid and robust bonding to bone, BG is widely used in bone regenerative procedures and for treating dentin hypersensitivity [12, 14, 15]. BG forms apatite, enhancing demineralized dentin's radiodensity and microhardness [16, 17, 18]. Wu et al. [12] reported that the dentin remineralization zone of BG is significantly larger than that of CPP-ACP and fluoride.

HA, particularly in its nano form (nHA), is renowned for promoting periodontal bone regeneration, reducing dentin hypersensitivity, and remineralizing carious lesions [19]. The morphology and crystalline structure of nHA resemble remineralization [20]. These nanoparticles can penetrate dentinal tubules, act as a scaffold during precipitation, and promote the integrity and growth of apatite crystals [21, 22]. They could also mechanically occupy the porous dental surface, potentially protecting the dentin from further demineralization [23]. A notable feature of HA is its capacity for ion substitution and inducing mineralization from the deeper levels of teeth, in contrast to fluoride, which mainly causes hypermineralization of the surface layers without strengthening the internal structure [24]. However, the effectiveness of nHA in remineralizing subsurface lesions and reducing the initiation or progression of initial caries lesions is still debated [25]. The biocompatibility, bioactivity, and mechanical strength of hydroxyapatite (HAp) are enhanced when combined with BG. The HAp-BG composite has been widely researched for its applications in bone tissue regeneration and repair [26]. However, the remineralizing efficacy of this composite for dental applications has yet to be as extensively studied. Notably, nHA, BG, and fluoride each have been shown to have a remineralizing effect at different depths [6, 7, 16, 21, 22]. Therefore, in this study, dentin-remineralizing gels containing varying concentrations of nHAp and BG, combined with 0.05 wt% sodium fluoride (NaF), were prepared, and their remineralization effects on dentin were evaluated at different depths. The findings of this study could be beneficial in remineralizing dentin carious lesions in patients with gingival recession or Class V primary lesions. Additionally, it provides an opportunity for future research to compare the effectiveness of available commercial remineralizing agents with the proposed combination from this study.

## 2. Materials and Methods

### 2.1. Sample Collection and Preparation

The protocol of this *in vitro* study was approved by the ethics committee of the Mashhad University of Medical Sciences (IR.MUMS.DENTISTRY.REC.1400.072).

According to the findings of a study by Fallahzadeh et al. [27], comparing the remineralizing efficacy of a BG composite with CPP-ACP and fluoride and considering alpha values of 0.05 and a beta of 0.20, 7 samples were calculated for each group, which was increased to 10 samples to increase the study power.

Forty intact bovine central incisors were gathered and placed in a 0.1% thymol solution for 1 week, after which they were stored in a normal saline solution. At the time of the study, the samples' roots were removed, and their crowns were mounted in self-curing acrylic (Acropars, Marlic Co., Tehran, Iran). The labial enamel layer was removed with a trimmer to expose the dentin [28]. Enamel removal was continued until dentinal tubules were observed through a stereomicroscope. Then, the dentin surfaces were polished using wet sandpapers (Starcke, Germany).

### 2.2. Gel Preparation

The experimental gel base was prepared by mixing 10 cc of glycerin with 10 cc of distilled water. At room temperature, three groups were created based on the added weight percentage of the experimental material. Since 0.05 wt% NaF (Merke, Darmstadt, Germany) is a well-proven cariostatic agent [29], it was included in the experimental groups. The gels were differentiated by the percentages of nHA (Pardis Pazhoohesh Co., Yazd, Iran) and 45S5 BG (Pardis Pazhoohesh Co., Yazd, Iran). According to a pilot experiment, the addition of more than 10 wt% powder caused the solution to lose its gel state. Subsequently, the 10% powder was divided between BG and nHA into three experimental groups:

- Group 1: 2.5 wt% nHA − 7.5 wt% 45S5 BG;
- Group 2: 5 wt% nHA − 5 wt% 45S5 BG;
- Group 3: 7.5 wt% nHA − 2.5 wt% 45S5 BG.

Then, 2 wt% carboxymethyl cellulose (CMC) (2 × 10–2 g/cc) was gradually added to the solution at 40–50°C to achieve the desired consistency. After 5 wt% silica (5 × 10–2 g/20 cc) was added, the mixture was stirred for 1–2 hr at 40–50°C. The gels were then refrigerated for 48 hr. The control group was treated with a gel base without a remineralizing agent.

### 2.3. Primary Carious Lesion Formation

A solution for demineralization was made by combining 2.2 mmol/L of CaCl_2_, 2.2 mmol/L of Na_2_HPO_4_, 50 mmol/L of acetic acid, and 0.2 mmol/L of sodium benzoate [5]. The pH of the solution was adjusted to 4.5 using 1 mol/L NaOH. The samples were demineralized for 8 hr at 37°C.

### 2.4. PH Cycling

The teeth were subjected to pH cycling for 20 days. First, the specimens' surfaces were dried, and a gel layer was applied to the tooth surface for 2 min. Then, all samples were placed in a remineralizing solution with the following composition for 1 hr: NaH_2_PO_4_ (0.033%), KCl (0.077%), CaCl_2_·2H_2_O (0.03%), MgCl_2_ (0.007%), NaHCO_3_ (0.0105%), sucrose (25 mL) at pH = 6.5). Another layer of remineralization agent was applied for 2 min, and samples were stored in the remineralization solution for another hour. Then, they were immersed in a demineralization solution for 4 hr. The samples were placed in the remineralization solution at room temperature for 1 hr. After two cycles of surface treatment with the gels, the samples were placed in the remineralization solution at room temperature for 16 hr.

### 2.5. Microhardness Measurement

Microhardness measurement was conducted following demineralization and after the pH cycling. A longitudinal section was made in the middle of each sample and polished with silicon carbide papers. Microhardness was measured using a Vickers microhardness tester (MH3, Koopa Pashoohesh, Iran) under 10 N loads applied for 10 s at three different points, each 1 mm apart at depths of 30, 60, and 140 *μ*m from the surface. The percentage surface microhardness recovery (SMHR%) was calculated via the following formulation [30]:

(1) $$\begin{array}{c} \text{SMHR}\%=\left(\frac{\text{microhardness after minerlization -microhardness after deminerlization}}{\text{baseline surface microhardness- microhardness after deminerlization}}\right)\times 100. \end{array}$$

### 2.6. Scanning Electron Microscopy (SEM) Analysis

Two specimens from each group were longitudinally split in half and were gold-palladium coated and analyzed through SEM (FEI-XL30, FEI Company, Hillsboro, Oregon, USA). The superficial morphology of each specimen was evaluated at 2,500× magnifications by two experts (S.M. and H.B.).

### 2.7. Statistical Analysis

Data were analyzed using Statistical Package for Social Sciences (SPSS) software version 29.0 (IBM Inc., Chicago, IL, USA). The Shapiro–Wilk test was used to assess the normal distribution of data. One-way ANOVA and Tukey post hoc tests were used to compare the SMHR% between the groups at different depths. The significance level was set at 0.05.

## 3. Results

Table 1 presents the average microhardness and SMHR% at various depths in intact, demineralized, and remineralized dentin. The microhardness of intact and demineralized dentin was comparable between the groups at all depths. At a depth of 30 *μ*m, the microhardness of remineralized dentin of the control group was lower than in Groups 1 (*p* = 0.043), 2 (*p*  < 0.001), and 3 (*p*  < 0.001). The Group 1 was also had lower microhardness than the Groups 2 (*p* = 0.007) and 3 (*p* = 0.001). At a depth of 60 *μ*m, the microhardness of the control group was lower than that of Groups 2 (*p* = 0.002) and 3 (*p*  < 0.001), and the microhardness of Group 3 was higher than Group 1 (*p* = 0.003). At a depth of 140 *μ*m, the microhardness of the remineralized dentin of the control group was lower than in Groups 2 (*p* = 0.007) and 3 (*p* = 0.001).

When the SMHR% was evaluated at a 30 *μ*m depth, the SMHR% of Group 2 (189.82% ± 51%) and Group 3 (187. 94% ± 68.95%) was significantly higher than the control group (*p*  < 0.001) and the Group 1 (*p* = 0.004 and *p* = 0.005, respectively). The SMHR% of Group 1 was significantly higher than that of the control group at this depth (*p* = 0.047).

At a 60 *μ*m depth, the SMHR% of Group 2 (152.14% ± 88.63%) and Group 3 (179.55% ± 75.96%) was significantly higher than that of the control group (*p* = 0.009 and *p* = 0.002, respectively). The SHMR% of Group 3 was significantly higher than that of Group 1 (*p* = 0.043). However, the SMHR% of Groups 1 and 2 and Group 1 and the control group were comparable.

At a 140 *μ*m depth, Group 2 (147.45% ± 85.30%) and Group 3 (175.93% ± 11.90%) had a significantly higher SMHR% than the control group (*p* = 0.011 and *p* = 0.004, respectively). However, the SMHR% of Group 1, the control group, and the SMHR% of Groups 1, 2, and 3 were comparable.

**Figure 1:**
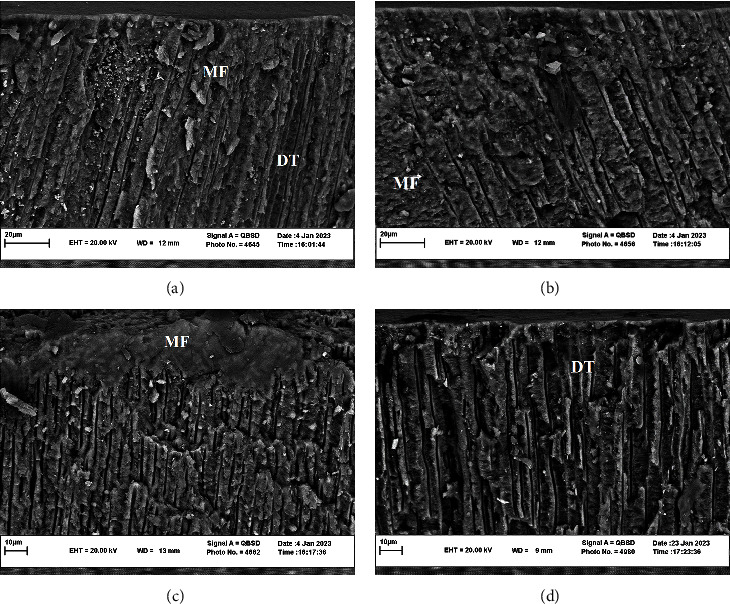
SEM imaged of the longitudinal section of dentin samples treated with (a) Group 1 (2.5% nHA + 7.5% BG + 0.05% NaF); (b) Group 2 (5% nHA + 5% BG + 0.05% NaF); (c) Group 3 (7.5% nHA + 2.5% BG + 0.05% NaF); (d) control group (gel base) at 2,500x magnifications. More minerals filling (MF) and precipitations can be observed in the dentin tubules (DT) in (b) and (c) groups, while in the control group, most of the tubules are completely or partially open.

Figure 1 represents the SEM images of the samples in the groups. Visible surface deposition and occlusion of tubules can be observed in the Groups 2 and 3 samples, which is more prominent in the latter (Figures 1(b) and 1(c)). Partial occlusion can be observed in the samples of Group 1. However, in the control group, most dentin tubules were open.

The effect size (Cohen's *d*) for the comparison between Group 1 and the Control at a depth of 30 *μ*m was ~2.11. With an alpha level of 0.05 and a sample size of 10 per group, the study power was calculated to be ~99.4%. This high power indicates a high probability of detecting a true effect if one exists, suggesting that the chosen sample size and observed effect size are sufficient to achieve a robust level of statistical significance.

## 4. Discussion

Our study's findings indicate that the groups with the highest concentration of nHA and similar concentrations of nHA and BG showed the most significant dentin microhardness recovery values, particularly at depths of 30 and 60 *µ*m. However, the microhardness recovery value of these two combinations was higher than the control group at all depths. Therefore, the null hypothesis was rejected.

In this in vitro study, bovine teeth were utilized. Using human teeth poses challenges, such as obtaining them in sufficient quantity and quality, as they are often extracted due to extensive carious lesions or other defects [31]. Additionally, the structure and composition of human teeth vary with age and individual differences, leading to variability in study results [20]. Bovine teeth are preferred among non-human dental hard tissues due to their availability, uniform composition, and similarity to human teeth, particularly in terms of calcium content [32].

Surface microhardness testing was selected for this study since it is simple, reliable, nondestructive, and quick [33]. Featherstone et al. [34] identified a correlation between microhardness values and mineral ratios in carious lesions. A distinctive aspect of the current study was measuring microhardness at three different depths. This approach contrasts with most other studies focusing on surface microhardness. Moreover, microhardness recovery was assessed to the depth of 140 *μ*m, which provides sufficient depth for assessing the remineralization [4].

An important point is that our experimental gels are not dentifrices used during toothbrushing. Instead, these gels are applied similarly to commercial MI Paste® (GC America, Illinois, United States). They should be applied directly to the affected tooth areas using a finger after oral hygiene and left on for 1–3 min [35]. Therefore, we applied the gels for 2 min. The pH cycling model we used was also designed to replicate the fluctuating mineral saturation and acidic challenges seen in natural caries formation [12].

Elasser et al. [17] noted that under both neutral and acidic conditions, nHA and nano-BG create denser dentin than NaF. However, the solubility dynamics of nHA and silica are pH-dependent, affecting precipitation [36]. By combining these two substances, our experimental gels could potentially more effectively remineralize carious lesions across varying depths in both acidic and neutral oral environments, compared to using each substance separately. Furthermore, the presence of a minimal fluoride concentration aids in penetrating other remineralizing agents [37]. Therefore, we added 0.05 wt% NaF to the experimental gels. About 0.05 wt% NaF is the concentration of daily fluoride mouthwash known to enhance carious lesion remineralization, has an antibacterial effect, and prevents WSLs formation and progression [11, 38].

The most notable microhardness recovery values were observed in the group treated with 7.5 wt% nHA, 2.5 wt% BG, and 0.05 wt% NaF at all depths. nHA is thought to deposit more minerals on the outer layer, potentially inhibiting mineral diffusion due to the highly mineralized surface layer [39]. However, initial caries lesions can be rehardened by HA, starting near the surface and gradually moving inward, ultimately precipitating in the dark zone during long-term remineralization. Regular use of nHA shows promising potential for promoting remineralization [21]. Additionally, incorporating fluoride enhances the penetration of other remineralizing agents. The SEM analysis also showed the most prominent deposition in this group, mostly on the outer surface of dentin; however, the tubules were mostly closed to their full length. Ebadifar et al. [40] found that a toothpaste containing 7% HA and 1,000 ppm NaF increased microhardness after acid exposure more than toothpaste with NaF alone, indicating a possible synergistic effect of HA and fluoride. However, Comar et al. [23] observed no positive effect on dentin remineralization when fluoride was added to 10% or 20% nHA. They also reported that 7-day applications of nHA pastes at various concentrations did not prevent demineralization, irrespective of lesion depth. This contrasting result to our study might be due to the longer duration of our pH cycling (20 days) and our focus on remineralization of artificial lesions rather than demineralization. Additionally, the presence of BG in our study could account for our findings. Conversely, Rodemer et al. [20] suggested that combining nHA with fluoride might inversely affect the stability and persistence of calcium fluoride surface precipitates. However, the differences in our results might be attributed to our distinct methodologies (pH cycling and microhardness assessment versus EDX and exposure to air and water syringe) and the higher nHA concentration in our study (7.5% versus 5%).

BG, a highly biocompatible calcium sodium phosphosilicate, initiates surface interactions in three stages: first, leaching and cation transfer (involving Si4+, OH−1, Na+, and Ca2+); secondly, the network disintegration of SiO_2_, leading to the precipitation of calcium-phosphate and crystallization into a carbonated HA layer; and finally, the occlusion of dentinal tubules [41, 42, 43]. However, the relatively large size of BG particles, ~5 *μ*m in our study, might have hindered their penetration into dentinal tubules. The smaller diameter of these tubules (less than 4 *μ*m) accounts for the lower microhardness values in samples with high BG content. Despite this, the strong bonding capacity of BG to the tooth structure and its role in occluding dentin tubules [12] contribute to the higher microhardness values observed in the experimental gel containing 7.5 wt% BG compared to the control samples.

Our study's gels show potential as remineralizing agents for treating dentin-carious lesions, such as root dentin in individuals with pronounced gingival recession or Class V primary lesions. They may also be potential agents for alleviating dentin sensitivity, as the SEM images showed that most dentin tubules were closed. However, it is important to note that in vitro studies like ours cannot fully replicate the complex nature of oral conditions. Previous research has shown that the pellicle can act as a bridge between nHA and dental materials, enhancing particle adhesion [44]. Therefore, further research is essential to assess the effectiveness of these experimental remineralization gels, particularly about their solubility in human saliva and dental pellicle. Additionally, a comparative analysis of the dentin remineralizing efficacy of our experimental gel with commercial gels or pastes containing other remineralizing agents would be beneficial.

## 5. Conclusion

The current study's findings suggested that the group with the highest nHA concentration exhibited the most favorable microhardness recovery values and dentin tubule closure from the depth of 30–140 *μ*m. Therefore, combining the remineralizing agents proposed in our study may hold promising potential for clinical applications in remineralizing primary dentin lesions.

## Figures and Tables

**Table 1:** Mean ± standard deviation (SD) of microhardness values of intact, demineralized, and remineralized dentin and microhardness recovery (%) of the study groups at different depths.

Depth	Groups	Composition (nHA–BG–NaF)	Intact dentin (mean ± SD)	Demineralized dentin (mean ± SD)	Remineneralized dentin (mean ± SD)	Microhardness recovery (%)
30 *μ*m	Group 1	2.5%–7.5%−0.05%	60.32 ± 11.29	37.40 ± 7.41	51.06 ± 8.96A	67.39 ± 29.34A
	Group 2	5.5%–5.5%−0.05%	55.030 ± 10.61	39.77 ± 12.92	68.59 ± 9.62B	189.82 ± 51B
	Group 3	7.5%–2.5%−0.05%	60.41 ± 6.34	42.45 ± 4.41	74.49 ± 7.96B	187.94 ± 68.95B
	Control	Gel base	58.34 ± 8.59	40.16 ± 7.80	36.37 ± 18.48C	−21.24 ± 51.72C
*p* Value			0.525	0.776	<0.001 ^*∗*^	<0.001 ^*∗*^

60 *μ*m	Group 1	2.5%–7.5%−0.05%	68.96 ± 16.37	49.16 ± 10.14	58.80 ± 7.94A	64.34 ± 41.96AB
	Group 2	5.5%–5.5%−0.05%	65.35 ± 11.66	50.30 ± 12.18	68.25 ± 10.45B	152.14 ± 88.63BC
	Group 3	7.5%–2.5%−0.05%	65.74 ± 3.80	51.62 ± 4.75	74.57 ± 5.76B	179.55 ± 75.96C
	Control	Gel base	69.61 ± 1.11	51.39 ± 8.22	51.72 ± 11.37C	8.71 ± 63.37A
*p* Value			0.798	0.962	<0.001 ^*∗*^	0.001 ^*∗*^

140 *μ*m	Group 1	2.5%–7.5%−0.05%	75.02 ± 12.61	61.20 ± 9.03	69.16 ± 9.42AB	59.90 ± 14.28AB
	Group 2	5.5%–5.5%−0.05%	71.48 ± 9.40	61.05 ± 9.15	72.81 ± 11.62A	147.45 ± 85.30A
	Group 3	7.5%–2.5%−0.05%	73.08 ± 3.86	63.33 ± 5.48	78.42 ± 5.31A	175.93 ± 11.90A
	Control	Gel base	63.26 ± 16.77	61.90 ± 6.00	54.55 ± 19.21B	−60.26 ± 19.59B
*p* Value			0.275	0.972	0.011^*∗*^	0.004^*∗*^

^*∗*^Values less than 0.05 represent a significant difference among the groups in each depth according to the ANOVA test. At each depth, different uppercase letters in each column represent significant differences among the groups according to the Tuckey post hoc test.

## Data Availability

The data that support the findings of this study are available from the corresponding author upon reasonable request.
